# Different lumbar fusion techniques for lumbar spinal stenosis: a Bayesian network meta-analysis

**DOI:** 10.1186/s12893-023-02242-w

**Published:** 2023-11-15

**Authors:** Wei Li, Haibin Wei, Ran Zhang

**Affiliations:** Department of Pain Treatment, Shunyi District Hospital of Beijing, Beijing, 101300 China

**Keywords:** Minimally invasive technique, Lumbar fusion, Lumbar spinal stenosis, Network meta-analysis

## Abstract

**Objective:**

To comprehensively compare and assess the effects of different lumbar fusion techniques in patients with lumbar spinal stenosis (LSS).

**Methods:**

PubMed, Embase, Cochrane Library, and Web of Science databases were systematically searched up to December 24, 2022 in this network meta-analysis. Outcomes were pain (pain, low back pain, and leg pain), Japanese Orthopaedic Association (JOA), Oswestry Disability Index (ODI), complications, reoperation, and fusion. Network plots illustrated the direct and indirect comparisons of different fusion techniques for the outcomes. League tables showed the comparisons of any two fusion techniques, based on both direct and indirect evidence. The efficacy of each fusion technique for LSS was ranked by rank probabilities.

**Results:**

Totally 29 studies involving 2,379 patients were eligible. For pain, percutaneous endoscopic transforaminal lumbar interbody fusion (Endo-TLIF) was most likely to be the best technique, followed by minimally invasive transforaminal lumbar interbody fusion (MIS-TLIF), extreme lateral interbody fusion (XLIF), and transforaminal lumbar interbody fusion (TLIF). Percutaneous endoscopic posterior lumbar interbody fusion (Endo-PLIF) had the greatest likelihood to be the optimal technique for low back pain, followed sequentially by MIS-TLIF, minimally invasive posterior lumbar interbody fusion (MIS-PLIF), XLIF, Endo-TLIF, TLIF, oblique lumbar interbody fusion (OLIF), posterior lumbar interbody fusion (PLIF), and posterolateral lumbar fusion (PLF). MIS-PLIF was ranked the most effective technique concerning leg pain, followed by Endo-TLIF, MIS-TLIF, TLIF, Endo-PLIF, PLIF, OLIF, PLF, and XLIF. As regards JOA scores, Endo-TLIF had the maximum probability to be the best technique, followed by MIS-TLIF and TLIF. Endo-PLIF had the greatest likelihood to be the optimum technique for complications, followed by TLIF, MIS-TLIF, Endo-TLIF, OLIF, and XLIF.

**Conclusion:**

Minimally invasive fusion techniques may be effective in the treatment of LSS, compared with traditional techniques. Minimally invasive techniques were likely non-inferior with regards to postoperative complications.

**Supplementary Information:**

The online version contains supplementary material available at 10.1186/s12893-023-02242-w.

## Introduction

Lumbar spinal stenosis (LSS) refers to “the narrowing of the spinal canal, lateral recesses, or intervertebral foramina, which may cause bone or soft tissue to compress nerve roots” in the lumbar spine [1]. This frequently occurring degenerative disease is characterized by pain and neurogenic claudication [2], resulting in spinal degeneration as individuals age [3]. LSS can lead to substantial pain and disability, and greatly reduce the quality of life [4]. Besides, it may increase the risk of cardiovascular and neurodegenerative diseases [5].

Surgical intervention is necessary when conservative treatment is not effective. Approximately 600,000 LSS surgeries are performed annually in the United States [6]. Posterolateral lumbar fusion (PLF), posterior lumbar interbody fusion (PLIF) and transforaminal lumbar interbody fusion (TLIF) are commonly used surgical approaches in the treatment of LSS [7–10]. With the development of minimally invasive surgery (MIS), MIS-TLIF has been reported to be a safe procedure with satisfactory outcomes and acceptable complications when compared with TLIF [11]. In recent years, percutaneous endoscopic lumbar interbody fusion (Endo-LIF), a new technology and a research hotspot, achieves less surgical trauma, improves surgical visualization, and enhances recovery after surgery [12, 13]. It was found that patients undergoing oblique lumbar interbody fusion (OLIF) had comparable clinical outcomes to those undergoing minimally invasive transforaminal lumbar interbody fusion (MIS-TLIF) [14]. Compared with open PLIF, percutaneous endoscopic posterior lumbar interbody fusion (Endo-PLIF) was less invasive and promoted postoperative recovery, despite longer operation time, as shown by a previous study [15]. Another study illustrated that minimally invasive posterior lumbar interbody fusion (MIS-PLIF) exhibited similar effects to PLIF on 1‐year surgical outcomes [Visual Analog Scale and Oswestry Disability Index (ODI)] [16]. At present, only head-to-head comparisons are performed among various fusion techniques, some fusion techniques are not compared directly, and the effects of different fusion techniques for LSS patients remain unclear, which requires a network meta-analysis for simultaneous comparison by considering direct and indirect evidence.

This network meta-analysis aimed to comprehensively compare and assess the effects of different lumbar fusion techniques on pain (pain, low back pain, and leg pain), Japanese Orthopaedic Association (JOA), ODI, complications, reoperation, and fusion in patients with LSS, using both direct and indirect evidence.

## Methods

### Search strategy

Relevant published studies were retrieved from PubMed, Embase, Cochrane Library, and Web of Science databases up to December 24, 2022. The comprehensive search was conducted by two reviewers independently, and they discussed with each other when disagreements arose. English search terms consisted of “lumbar spinal stenosis” AND “LSS” AND “spinal stenosis” AND “degenerative disease of the lumbar spine” AND “lumbar degenerative disease” AND “spondylolisthesis” AND “lumbar fusion” AND “spinal fusion” AND “anterior lumbar interbody fusion” AND “ALIF” AND “posterior lumbar fusion” AND “posterolateral lumbar fusion” AND “PLF” AND “posterior lumbar interbody fusion” AND “PLIF” AND “transforaminal lumbar interbody fusion” AND “TLIF” AND “lateral interbody fusion” AND “LLIF” AND “lateral lumbar interbody fusion” AND “extreme lateral interbody fusion” AND “XLIF” AND “direct lateral interbody fusion” AND “DLIF” AND “transpsoas lumbar interbody fusion” AND “trans-psoas lumbar interbody fusion” AND “oblique lumbar interbody fusion” AND “OLIF” AND “minimally invasive transforaminal lumbar interbody fusion” AND “MIS-TLIF”. Endnote X9 (Clarivate Analytics) was applied for primary screening, based on titles and abstracts. Subsequently, full texts were read to select eligible studies. This Bayesian network meta-analysis was performed in accordance with the Preferred Reporting Items for Systematic reviews and Meta-Analyses (PRISMA) guidelines.

## Study selection

The inclusion criteria were as follows: (1) studies on LSS patients with fusion levels ≤ 3; (2) studies comparing at least two of different lumbar fusion techniques for spinal level L3-L5: PLF, PLIF, TLIF, minimally invasive posterolateral lumbar fusion (MIS-PLF), MIS-PLIF, MIS-TLIF, extreme lateral interbody fusion (XLIF), OLIF, Endo-PLIF, percutaneous endoscopic transforaminal lumbar interbody fusion (Endo-TLIF), and circumferential fusion; (3) studies on at least one of the following outcomes: pain (pain, low back pain, leg pain) scores, JOA scores, ODI scores, complications, reoperation, and fusion; and (4) randomized controlled trials (RCTs) or cohort studies.

The exclusion criteria were as follows: (1) studies which had incomplete data or whose data could not be extracted; (2) animal experiments; (3) case reports, meeting aibstracts, letters, reviews, meta-analyses; or (4) studies not published in English.

## Data extraction

Two reviewers (HW and RZ) independently extracted data from the qualified studies. The data included the first author, year of publication, country, study design, population, group, sample size (N), sex (male/female), age (years), body mass index (BMI, kg/m^2^), spinal level, fusion level, follow-up time (FU), quality assessment (QA), and outcome. A third author (WL) resolved the differences that arose.

## Quality assessment

The quality of RCTs was assessed using the modified Jadad scale [17] in terms of random sequence generation, randomization concealment, blinding, and withdrawals and dropouts, with 1–3 as low quality and 4–7 as high quality. The Newcastle–Ottawa scale (NOS) [18] was applied for the quality evaluation of cohort studies based on study population selection, inter-group comparability and outcome measurement, with 0–3 as poor quality, 4–6 as fair quality, and 7–9 as good quality.

## Statistical analysis

This network meta-analysis was conducted using a Bayesian framework and a Monte Carlo Markov Chain (MCMC) model. The number of model chains was 4, the number of initial iterations was 20,000, the number of updated iterations was 50,000, and the step size was 1. Heterogeneity indicated the overall degree of difference in the same pair of comparisons, with the I^2^ statistic < 25% as low heterogeneity, 25–50% as moderate heterogeneity, and > 50% as high heterogeneity. Consistency referred to the statistical consistency between direct and indirect effect sizes for the same comparison. The deviation information criterions (DICs) of the consistency model and the non-consistency model were compared, and a smaller difference suggested a better fit. The absolute value of the difference in the DICs within 5 denoted consistency between indirect and direct evidence. Compared with a frequentist network meta-analysis, a Bayesian network meta-analysis has the following advantages: (1) a Bayesian approach can not only effectively integrate data and flexibly build models, but also use the obtained posterior probability to rank all interventions participating in the comparison and distinguish comparative advantages and disadvantages, while a frequentist method can only rely on the effect size and its 95% confidence interval (CI) obtained by pairwise comparison in ranking; and (2) since a frequentist approach uses the maximum likelihood method in parameter estimation, which estimates the maximum likelihood function through continuous iteration, it is prone to instability and biased results, while a Bayesian approach does not have this problem, so its estimated values are more accurate than those of a frequentist approach [19].

Network plots illustrated the direct and indirect comparisons of different fusion techniques for the outcomes. For pain, JOA scores and ODI scores, weighted mean differences (WMDs) and 95% credibility intervals (CrIs) were shown; for complications, reoperation and fusion rates, relative risks (RRs) and 95%CrIs were reported. WMDs or RRs and 95%CrIs of all direct and indirect comparisons were presented in forest plots. League tables presented the comparisons of any two fusion techniques, based on both direct and indirect evidence. Through rank probabilities, the efficacy of each fusion technique for LSS was exhibited and ranked. Statistically significant differences (i.e. WMDs/RRs/CrIs) for the comparison of therapeutic effects of different lumbar fusion approaches indicated that one fusion technique was significantly more effective than another fusion technique. Rank probabilities illustrated the comparative advantages of fusion approaches by ranking these approaches from the highest priority to lowest priority, regardless of whether there were statistically significant differences in therapeutic effects between various methods [20]. Statistical analysis was performed using STATA 15.1 (Stata Corporation, College Station, TX, USA) and R 4.1.3 (R Foundation for Statistical Computing, Vienna, Austria).

## Results

### Study characteristics

**Fig. 1:**
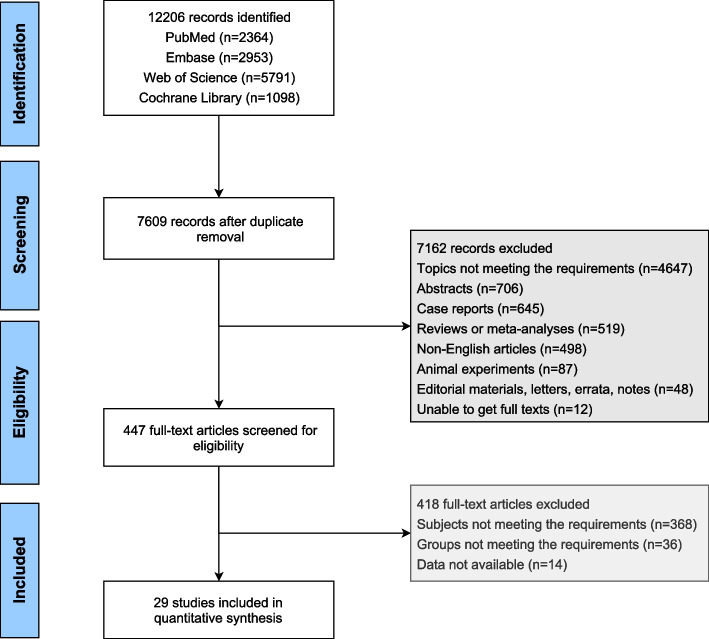
Flow chart of study selection

A total of 12,206 studies were identified from PubMed (*n* = 2,364), Embase (*n* = 2,953), Web of Science (*n* = 5,791), and Cochrane Library (*n* = 1,098). After duplicate removal, 7,609 studies were screened based on titles and abstracts, and then 447 studies were used for full-text screening. In the end, 29 studies [13, 15, 21–47] involving 2,379 patients were eligible for this network meta-analysis. Figure 1 describes the process of study selection. Of these included studies, 27 were cohort studies, with 10 of fair quality and 17 of good quality; 2 were RCTs, with 1 of low quality and 1 of high quality. Eleven fusion techniques were involved: PLF for 103 patients, PLIF for 283 patients, TLIF for 545 patients, MIS-PLF for 43 patients, MIS-PLIF for 67 patients, MIS-TLIF for 724 patients, XLIF for 152 patients, OLIF for 185 patients, Endo-PLIF for 69 patients, Endo-TLIF for 175 patients, and circumferential fusion for 33 patients. The year of publication ranged from 2007 to 2022. Baseline characteristics of the included studies are shown in Supplementary Table 1.

## Network meta-analysis for pain

### Pain

**Fig. 2:**
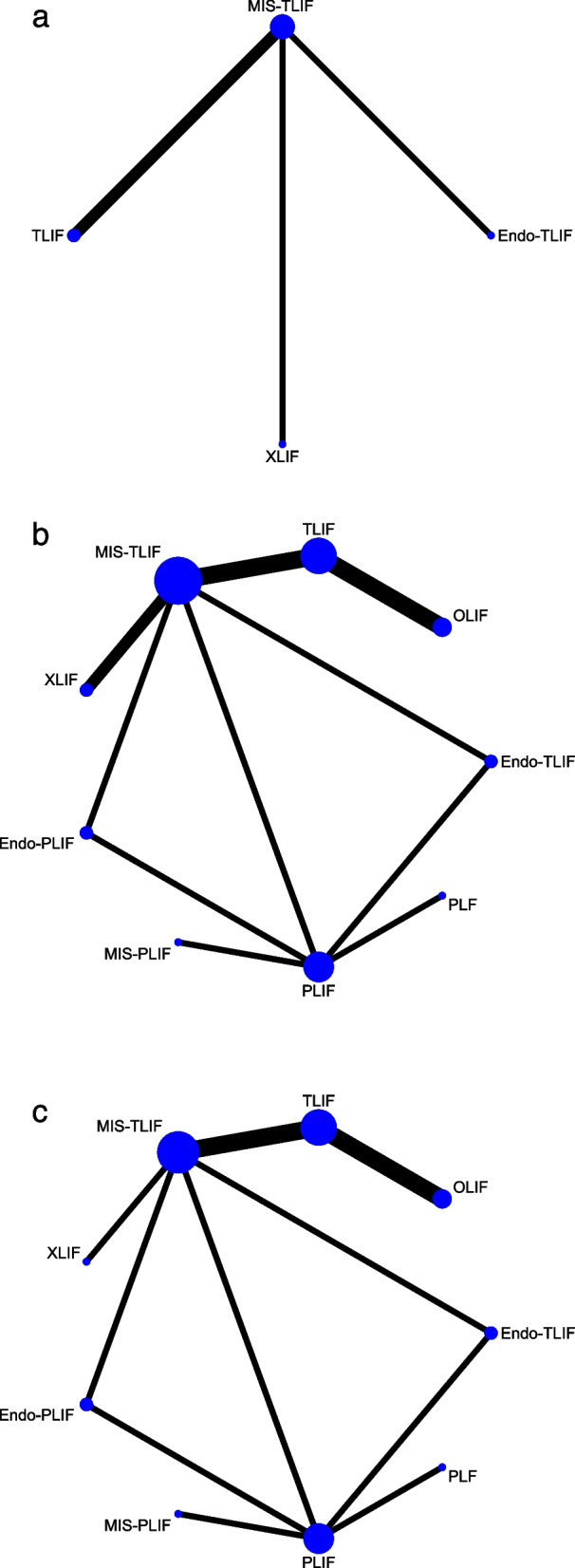
Network plots of different lumbar fusion techniques for pain in LSS. **a** pain; **b** low back pain; **c** leg pain. LSS, lumbar spinal stenosis; PLF, posterolateral lumbar fusion; PLIF, posterior lumbar interbody fusion; TLIF, transforaminal lumbar interbody fusion; MIS-PLIF, minimally invasive posterior lumbar interbody fusion; MIS-TLIF, minimally invasive transforaminal lumbar interbody fusion; XLIF, extreme lateral interbody fusion; OLIF, oblique lumbar interbody fusion; Endo-PLIF, percutaneous endoscopic posterior lumbar interbody fusion; Endo-TLIF, percutaneous endoscopic transforaminal lumbar interbody fusion

**Fig. 3:**
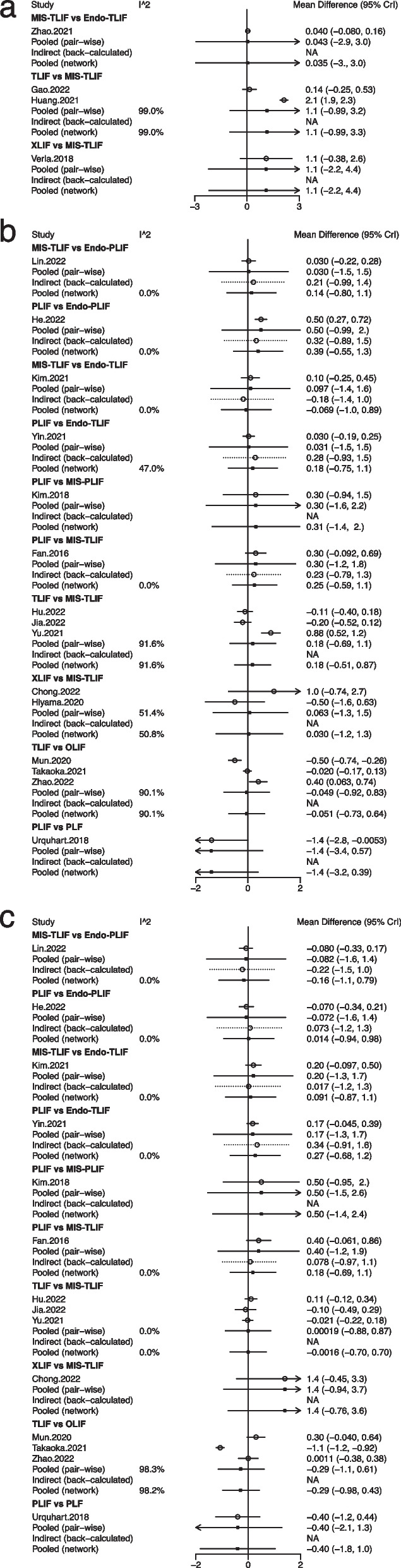
Forest plots of different lumbar fusion techniques for pain in LSS. **a** pain; **b** low back pain; **c** leg pain. LSS, lumbar spinal stenosis; PLF, posterolateral lumbar fusion; PLIF, posterior lumbar interbody fusion; TLIF, transforaminal lumbar interbody fusion; MIS-PLIF, minimally invasive posterior lumbar interbody fusion; MIS-TLIF, minimally invasive transforaminal lumbar interbody fusion; XLIF, extreme lateral interbody fusion; OLIF, oblique lumbar interbody fusion; Endo-PLIF, percutaneous endoscopic posterior lumbar interbody fusion; Endo-TLIF, percutaneous endoscopic transforaminal lumbar interbody fusion; CrI, credibility interval

Four studies with 245 patients provided data on pain, involving 4 fusion techniques: Endo-TLIF, MIS-TLIF, TLIF, and XLIF (Fig. 2a). No significant differences were observed in pain between MIS-TLIF and Endo-TLIF, between TLIF and MIS-TLIF, and between XLIF and MIS-TLIF in the forest plot (Fig. 3a). According to the league table, comparable pain scores were shown in patients undergoing any two of the fusion techniques (Table 1). The rank probabilities illustrated that for pain, Endo-TLIF was most likely to be the best technique, followed by MIS-TLIF, XLIF and TLIF (Table 2).

**Table 1:** League tables of different lumbar fusion techniques for outcomes in LSS

Pain											
	Endo-TLIF	MIS-TLIF	TLIF	XLIF							
Endo-TLIF	Endo-TLIF	0.04 (-2.95, 3.02)	1.18 (-2.49, 4.83)	1.15 (-3.30, 5.60)							
MIS-TLIF	-0.04 (-3.02, 2.95)	MIS-TLIF	1.14 (-0.99, 3.25)	1.11 (-2.19, 4.39)							
TLIF	-1.18 (-4.83, 2.49)	-1.14 (-3.25, 0.99)	TLIF	-0.03 (-3.96, 3.90)							
XLIF	-1.15 (-5.60, 3.30)	-1.11 (-4.39, 2.19)	0.03 (-3.90, 3.96)	XLIF							
Low back pain											
	Endo-PLIF	Endo-TLIF	MIS-PLIF	MIS-TLIF	OLIF	PLF	PLIF	TLIF	XLIF		
Endo-PLIF	Endo-PLIF	0.21 (-0.98, 1.38)	0.09 (-1.82, 2.01)	0.14 (-0.80, 1.08)	0.37 (-0.97, 1.72)	1.79 (-0.23, 3.79)	0.39 (-0.55, 1.33)	0.32 (-0.84, 1.49)	0.17 (-1.37, 1.77)		
Endo-TLIF	-0.21 (-1.38, 0.98)	Endo-TLIF	-0.12 (-2.03, 1.81)	-0.07 (-1.01, 0.89)	0.16 (-1.18, 1.53)	1.58 (-0.43, 3.58)	0.18 (-0.75, 1.12)	0.11 (-1.04, 1.29)	-0.04 (-1.57, 1.58)		
MIS-PLIF	-0.09 (-2.01, 1.82)	0.12 (-1.81, 2.03)	MIS-PLIF	0.05 (-1.83, 1.92)	0.28 (-1.84, 2.39)	1.69 (-0.75, 4.14)	0.31 (-1.38, 1.97)	0.23 (-1.78, 2.23)	0.08 (-2.18, 2.38)		
MIS-TLIF	-0.14 (-1.08, 0.80)	0.07 (-0.89, 1.01)	-0.05 (-1.92, 1.83)	MIS-TLIF	0.23 (-0.74, 1.20)	1.64 (-0.33, 3.61)	0.25 (-0.60, 1.10)	0.18 (-0.51, 0.87)	0.03 (-1.21, 1.32)		
OLIF	-0.37 (-1.72, 0.97)	-0.16 (-1.53, 1.18)	-0.28 (-2.39, 1.84)	-0.23 (-1.20, 0.74)	OLIF	1.42 (-0.78, 3.60)	0.02 (-1.27, 1.30)	-0.05 (-0.73, 0.64)	-0.20 (-1.76, 1.42)		
PLF	-1.79 (-3.79, 0.23)	-1.58 (-3.58, 0.43)	-1.69 (-4.14, 0.75)	-1.64 (-3.61, 0.33)	-1.42 (-3.60, 0.78)	PLF	-1.39 (-3.18, 0.39)	-1.46 (-3.54, 0.63)	-1.61 (-3.93, 0.76)		
PLIF	-0.39 (-1.33, 0.55)	-0.18 (-1.12, 0.75)	-0.3 (-1.97, 1.38)	-0.25 (-1.10, 0.60)	-0.02 (-1.30, 1.27)	1.39 (-0.39, 3.18)	PLIF	-0.07 (-1.16, 1.03)	-0.22 (-1.71, 1.33)		
TLIF	-0.32 (-1.49, 0.84)	-0.11 (-1.29, 1.04)	-0.23 (-2.23, 1.78)	-0.18 (-0.88, 0.51)	0.05 (-0.64, 0.73)	1.46 (-0.63, 3.54)	0.07 (-1.03, 1.16)	TLIF	-0.15 (-1.56, 1.31)		
XLIF	-0.17 (-1.77, 1.37)	0.04 (-1.58, 1.57)	-0.08 (-2.38, 2.18)	-0.03 (-1.32, 1.21)	0.20 (-1.42, 1.76)	1.61 (-0.76, 3.93)	0.22 (-1.33, 1.71)	0.15 (-1.31, 1.56)	XLIF		
Leg pain											
	Endo-PLIF	Endo-TLIF	MIS-PLIF	MIS-TLIF	OLIF	PLF	PLIF	TLIF	XLIF		
Endo-PLIF	Endo-PLIF	-0.25 (-1.46, 0.96)	-0.49 (-2.56, 1.60)	-0.16 (-1.12, 0.79)	0.13 (-1.27, 1.48)	0.41 (-1.3, 2.14)	0.01 (-0.94, 0.98)	-0.16 (-1.35, 1.01)	1.24 (-1.15, 3.61)		
Endo-TLIF	0.25 (-0.96, 1.46)	Endo-TLIF	-0.24 (-2.30, 1.84)	0.09 (-0.87, 1.05)	0.39 (-1.01, 1.73)	0.67 (-1.04, 2.39)	0.27 (-0.68, 1.23)	0.09 (-1.10, 1.27)	1.49 (-0.90, 3.86)		
MIS-PLIF	0.49 (-1.60, 2.56)	0.24 (-1.84, 2.30)	MIS-PLIF	0.33 (-1.72, 2.35)	0.62 (-1.66, 2.85)	0.90 (-1.42, 3.25)	0.50 (-1.35, 2.35)	0.33 (-1.83, 2.46)	1.73 (-1.26, 4.70)		
MIS-TLIF	0.16 (-0.79, 1.12)	-0.09 (-1.05, 0.87)	-0.33 (-2.35, 1.72)	MIS-TLIF	0.29 (-0.72, 1.26)	0.58 (-1.09, 2.25)	0.18 (-0.69, 1.05)	0.00 (-0.70, 0.70)	1.40 (-0.76, 3.57)		
OLIF	-0.13 (-1.48, 1.27)	-0.39 (-1.73, 1.01)	-0.62 (-2.85, 1.66)	-0.29 (-1.26, 0.72)	OLIF	0.29 (-1.62, 2.26)	-0.12 (-1.40, 1.23)	-0.29 (-0.98, 0.43)	1.11 (-1.28, 3.49)		
PLF	-0.41 (-2.14, 1.3)	-0.67 (-2.39, 1.04)	-0.90 (-3.25, 1.42)	-0.58 (-2.25, 1.09)	-0.29 (-2.26, 1.62)	PLF	-0.40 (-1.83, 1.03)	-0.58 (-2.40, 1.23)	0.82 (-1.94, 3.56)		
PLIF	-0.01 (-0.98, 0.94)	-0.27 (-1.23, 0.68)	-0.51 (-2.35, 1.35)	-0.18 (-1.05, 0.68)	0.12 (-1.23, 1.40)	0.40 (-1.03, 1.83)	PLIF	-0.18 (-1.30, 0.93)	1.22 (-1.14, 3.55)		
TLIF	0.16 (-1.01, 1.35)	-0.09 (-1.27, 1.10)	-0.33 (-2.46, 1.83)	0.00 (-0.70, 0.69)	0.29 (-0.43, 0.98)	0.58 (-1.23, 2.40)	0.18 (-0.93, 1.30)	TLIF	1.40 (-0.90, 3.68)		
XLIF	-1.24 (-3.61, 1.15)	-1.49 (-3.86, 0.90)	-1.73 (-4.70, 1.26)	-1.4 (-3.57, 0.79)	-1.11 (-3.49, 1.28)	-0.82 (-3.56, 1.94)	-1.22 (-3.55, 1.14)	-1.40 (-3.68, 0.90)	XLIF		
JOA											
	Endo-TLIF	MIS-TLIF	TLIF								
Endo-TLIF	Endo-TLIF	-0.19 (-8.42, 8.00)	-1.77 (-11.31, 7.70)								
MIS-TLIF	0.18 (-8.00, 8.42)	MIS-TLIF	-1.58 (-6.41, 3.19)								
TLIF	1.77 (-7.70, 11.31)	1.58 (-3.19, 6.41)	TLIF								
ODI											
	circumferential	Endo-PLIF	Endo-TLIF	MIS-PLIF	MIS-TLIF	OLIF	PLF	PLIF	TLIF	XLIF	
circumferential	circumferential	10.13 (-2.28, 22.64)	9.26 (-3.21, 21.86)	7.24 (-6.95, 21.45)	9.13 (-3.24, 21.62)	11.21 (-1.26, 23.80)	6.41 (-1.60, 14.37)	8.80 (-3.56, 21.28)	10.9 (-1.54, 23.44)	9.22 (-4.85, 23.33)	
Endo-PLIF	-10.13 (-22.64, 2.28)	Endo-PLIF	-0.87 (-2.97, 1.21)	-2.91 (-9.98, 4.08)	-1.01 (-2.01, -0.004)	1.08 (-0.85, 3.02)	-3.73 (-13.35, 5.80)	-1.33 (-2.37, -0.29)	0.77 (-0.81, 2.35)	-0.92 (-7.62, 5.81)	
Endo-TLIF	-9.26 (-21.86, 3.21)	0.87 (-1.21, 2.97)	Endo-TLIF	-2.04 (-9.28, 5.13)	-0.14 (-2.01, 1.74)	1.95 (-0.56, 4.46)	-2.85 (-12.60, 6.78)	-0.46 (-2.27, 1.36)	1.64 (-0.59, 3.88)	-0.05 (-6.94, 6.86)	
MIS-PLIF	-7.24 (-21.45, 6.95)	2.91 (-4.08, 9.98)	2.04 (-5.13, 9.28)	MIS-PLIF	1.91 (-5.04, 8.92)	3.99 (-3.18, 11.2)	-0.82 (-12.61, 10.91)	1.59 (-5.35, 8.54)	3.68 (-3.38, 10.81)	2.00 (-7.66, 11.67)	
MIS-TLIF	-9.13 (-21.62, 3.24)	1.01 (0.00, 2.01)	0.14 (-1.74, 2.01)	-1.91 (-8.92, 5.04)	MIS-TLIF	2.09 (0.43, 3.74)	-2.72 (-12.31, 6.75)	-0.32 (-0.86, 0.22)	1.78 (0.57, 3.00)	0.07 (-6.55, 6.74)	
OLIF	-11.21 (-23.8, 1.26)	-1.08 (-3.02, 0.85)	-1.95 (-4.46, 0.56)	-3.99 (-11.20, 3.18)	-2.09 (-3.74, -0.43)	OLIF	-4.80 (-14.53, 4.80)	-2.41 (-4.15, -0.66)	-0.31 (-1.44, 0.82)	-2.00 (-8.85, 4.85)	
PLF	-6.41 (-14.37, 1.50)	3.73 (-5.80, 13.35)	2.85 (-6.78, 12.60)	0.82 (-10.91, 12.61)	2.72 (-6.75, 12.31)	4.80 (-4.80, 14.53)	PLF	2.40 (-7.07, 11.97)	4.50 (-5.03, 14.17)	2.80 (-8.76, 14.46)	
PLIF	-8.80 (-21.28, 3.56)	1.33 (0.29, 2.37)	0.46 (-1.36, 2.27)	-1.59 (-8.57, 5.34)	0.32 (-0.22, 0.86)	2.41 (0.66, 4.15)	-2.40 (-11.97, 7.07)	PLIF	2.10 (0.76, 3.43)	0.40 (-6.24, 7.09)	
TLIF	-10.90 (-23.44, 1.54)	-0.77 (-2.35, 0.81)	-1.64 (-3.88, 0.59)	-3.68 (-10.81, 3.38)	-1.78 (-3.00, -0.55)	0.31 (-0.82, 1.44)	-4.50 (-14.17, 5.03)	-2.10 (-3.43, -0.76)	TLIF	-1.69 (-8.43, 5.06)	
XLIF	-9.22 (-23.33, 4.85)	0.92 (-5.81, 7.62)	0.05 (-6.86, 6.94)	-2.00 (-11.67, 7.66)	-0.09 (-6.74, 6.54)	2.00 (-4.85, 8.85)	-2.80 (-14.46, 8.76)	-0.40 (-7.09, 6.24)	1.69 (-5.06, 8.43)	XLIF	
Complications											
	Endo-PLIF	Endo-TLIF	MIS-TLIF	OLIF	TLIF	XLIF					
Endo-PLIF	Endo-PLIF	2.19 (0.08, 92.77)	2.02 (0.16, 56.09)	2.71 (0.18, 85.61)	1.82 (0.13, 55.45)	6.63 (0.47, 200.63)					
Endo-TLIF	0.46 (0.01, 12.11)	Endo-TLIF	0.93 (0.15, 7.68)	1.24 (0.16, 12.20)	0.83 (0.11, 7.82)	3.03 (0.41, 28.48)					
MIS-TLIF	0.50 (0.02, 6.16)	1.08 (0.13, 6.75)	MIS-TLIF	1.34 (0.52, 3.37)	0.89 (0.39, 2.00)	3.19 (1.57, 7.63)					
OLIF	0.37 (0.01, 5.48)	0.80 (0.08, 6.35)	0.75 (0.30, 1.92)	OLIF	0.67 (0.43, 1.05)	2.42 (0.74, 8.53)					
TLIF	0.55 (0.02, 7.94)	1.20 (0.13, 9.00)	1.12 (0.50, 2.55)	1.49 (0.95, 2.36)	TLIF	3.59 (1.21, 11.8)					
XLIF	0.15 (0.00, 2.12)	0.33 (0.04, 2.41)	0.31 (0.13, 0.64)	0.41 (0.12, 1.35)	0.28 (0.08, 0.83)	XLIF					
Reoperation											
	circumferential	Endo-TLIF	MIS-TLIF	OLIF	PLF	PLIF	TLIF	XLIF			
circumferential	circumferential	9.59 (0.02, 7583.09)	5.39 (0.04, 1658.52)	15.55 (0.09, 5842.20)	0.88 (0.02, 31.39)	4.01 (0.06, 463.97)	12.92 (0.09, 3819.25)	3.20 (0.01, 1277.30)			
Endo-TLIF	0.10 (0.00, 45.92)	Endo-TLIF	0.58 (0.02, 20.38)	1.66 (0.03, 89.10)	0.09 (0.00, 12.96)	0.45 (0.00, 36.64)	1.38 (0.03, 50.18)	0.34 (0.01, 17.16)			
MIS-TLIF	0.19 (0.00, 26.68)	1.74 (0.05, 65.92)	MIS-TLIF	2.90 (0.44, 18.73)	0.17 (0.00, 5.21)	0.83 (0.03, 11.03)	2.41 (1.19, 4.84)	0.62 (0.08, 3.24)			
OLIF	0.06 (0.00, 11.71)	0.6 (0.01, 35.12)	0.35 (0.05, 2.25)	OLIF	0.06 (0.00, 2.56)	0.28 (0.01, 5.94)	0.83 (0.15, 4.74)	0.21 (0.01, 2.61)			
PLF	1.15 (0.03, 41.16)	10.63 (0.08, 3417.14)	5.75 (0.19, 644.62)	16.97 (0.39, 2320.71)	PLF	4.01 (0.63, 102.71)	13.84 (0.51, 1513.92)	3.52 (0.07, 523.86)			
PLIF	0.25 (0.00, 15.88)	2.24 (0.03, 302.84)	1.21 (0.09, 39.44)	3.59 (0.17, 157.55)	0.24 (0.01, 1.61)	PLIF	2.89 (0.24, 89.89)	0.75 (0.03, 34.19)			
TLIF	0.08 (0.00, 10.55)	0.72 (0.02, 28.76)	0.42 (0.21, 0.84)	1.20 (0.21, 6.79)	0.07 (0.00, 1.96)	0.35 (0.01, 4.15)	TLIF	0.26 (0.03, 1.55)			
XLIF	0.31 (0.00, 68.28)	2.92 (0.06, 181.69)	1.61 (0.31, 12.82)	4.78 (0.38, 74.62)	0.28 (0.00, 15.02)	1.34 (0.03, 36.74)	3.91 (0.65, 34.72)	XLIF			
Fusion											
	circumferential	Endo-PLIF	Endo-TLIF	MIS-PLF	MIS-PLIF	MIS-TLIF	OLIF	PLF	PLIF	TLIF	XLIF
circumferential	circumferential	0.85 (0.62, 1.14)	0.92 (0.69, 1.22)	0.03 (0.00, 0.76)	0.96 (0.72, 1.27)	0.92 (0.68, 1.23)	0.94 (0.69, 1.28)	0.91 (0.72, 1.09)	0.96 (0.74, 1.25)	0.95 (0.71, 1.27)	1.00 (0.74, 1.36)
Endo-PLIF	1.18 (0.87, 1.62)	Endo-PLIF	1.07 (0.96, 1.30)	0.03 (0.00, 0.90)	1.12 (0.95, 1.38)	1.07 (0.97, 1.29)	1.1 (0.95, 1.36)	1.07 (0.85, 1.35)	1.13 (0.99, 1.36)	1.11 (0.99, 1.35)	1.17 (1.02, 1.44)
Endo-TLIF	1.09 (0.82, 1.45)	0.93 (0.77, 1.04)	Endo-TLIF	0.03 (0.00, 0.83)	1.04 (0.90, 1.21)	1.00 (0.94, 1.07)	1.02 (0.91, 1.15)	0.99 (0.79, 1.19)	1.04 (0.94, 1.17)	1.03 (0.95, 1.13)	1.08 (0.98, 1.23)
MIS-PLF	35.65 (1.31, 113,395.97)	30.19 (1.11, 94,731.25)	32.68 (1.21, 102,493.79)	MIS-PLF	34.11 (1.26, 106,641.47)	32.63 (1.20, 102,391.47)	33.47 (1.23, 104,609.18)	33.28 (1.20, 101,428.96)	34.19 (1.26, 106,953.77)	33.78 (1.25, 105,413.31)	35.63 (1.31, 111,795.43)
MIS-PLIF	1.04 (0.79, 1.39)	0.89 (0.72, 1.05)	0.96 (0.83, 1.11)	0.03 (0.00, 0.79)	MIS-PLIF	0.96 (0.82, 1.12)	0.98 (0.82, 1.18)	0.95 (0.77, 1.13)	1.00 (0.91, 1.11)	0.99 (0.84, 1.17)	1.04 (0.87, 1.25)
MIS-TLIF	1.09 (0.82, 1.46)	0.94 (0.77, 1.03)	1.00 (0.94, 1.07)	0.03 (0.00, 0.83)	1.05 (0.9, 1.22)	MIS-TLIF	1.03 (0.92, 1.13)	1.00 (0.79, 1.20)	1.05 (0.93, 1.19)	1.03 (0.99, 1.09)	1.09 (1.01, 1.21)
OLIF	1.06 (0.78, 1.45)	0.91 (0.74, 1.06)	0.98 (0.87, 1.10)	0.03 (0.00, 0.81)	1.02 (0.85, 1.23)	0.97 (0.88, 1.08)	OLIF	0.97 (0.76, 1.20)	1.02 (0.87, 1.20)	1.01 (0.93, 1.11)	1.06 (0.93, 1.23)
PLF	1.09 (0.91, 1.38)	0.93 (0.74, 1.18)	1.01 (0.84, 1.26)	0.03 (0.00, 0.84)	1.05 (0.89, 1.30)	1.00 (0.83, 1.26)	1.03 (0.83, 1.32)	PLF	1.05 (0.92, 1.28)	1.04 (0.86, 1.31)	1.1 (0.89, 1.40)
PLIF	1.04 (0.8, 1.36)	0.89 (0.74, 1.01)	0.96 (0.85, 1.07)	0.03 (0.00, 0.79)	1.00 (0.90, 1.09)	0.95 (0.84, 1.08)	0.98 (0.83, 1.15)	0.95 (0.78, 1.09)	PLIF	0.99 (0.87, 1.13)	1.04 (0.89, 1.22)
TLIF	1.05 (0.78, 1.42)	0.90 (0.74, 1.01)	0.97 (0.89, 1.05)	0.03 (0.00, 0.80)	1.01 (0.86, 1.19)	0.97 (0.91, 1.01)	0.99 (0.90, 1.08)	0.96 (0.76, 1.17)	1.01 (0.89, 1.16)	TLIF	1.05 (0.95, 1.18)
XLIF	1 (0.74, 1.36)	0.86 (0.69, 0.98)	0.92 (0.81, 1.02)	0.03 (0.00, 0.76)	0.96 (0.80, 1.14)	0.92 (0.82, 1.00)	0.94 (0.81, 1.07)	0.91 (0.71, 1.12)	0.96 (0.82, 1.12)	0.95 (0.85, 1.05)	XLIF

*LSS* lumbar spinal stenosis, *JOA* Japanese Orthopaedic Association, *ODI* Oswestry Disability Index, *PLF* posterolateral lumbar fusion, *PLIF* posterior lumbar interbody fusion, *TLIF* transforaminal lumbar interbody fusion, *MIS-PLF* minimally invasive posterolateral lumbar fusion, *MIS-PLIF* minimally invasive posterior lumbar interbody fusion, *MIS-TLIF* minimally invasive transforaminal lumbar interbody fusion, *XLIF* extreme lateral interbody fusion, *OLIF* oblique lumbar interbody fusion, *Endo-PILF* percutaneous endoscopic posterior lumbar interbody fusion, *Endo-TILF* percutaneous endoscopic transforaminal lumbar interbody fusion

**Table 2:** Rank probabilities of different lumbar fusion techniques for outcomes in LSS

Pain											
	[1]	[2]	[3]	[4]							
Endo-TLIF	0.1285	0.19216	0.23194	0.4474							
MIS-TLIF	0.01624	0.167745	0.489285	0.32673							
TLIF	0.4174	0.380275	0.137635	0.06469							
XLIF	0.43786	0.25982	0.14114	0.16118							
Low back pain											
	[1]	[2]	[3]	[4]	[5]	[6]	[7]	[8]	[9]		
Endo-PLIF	0.005615	0.03354	0.054395	0.07769	0.09583	0.116445	0.16064	0.240805	0.21504		
Endo-TLIF	0.014215	0.08987	0.118995	0.13434	0.13327	0.133105	0.141095	0.141905	0.093205		
MIS-PLIF	0.055935	0.178515	0.081055	0.06635	0.057925	0.05672	0.069275	0.115285	0.31894		
MIS-TLIF	0.00133	0.01221	0.04199	0.10473	0.183265	0.23769	0.23172	0.145205	0.04186		
OLIF	0.0437	0.188985	0.171485	0.137805	0.118425	0.100235	0.090235	0.086055	0.063075		
PLF	0.810125	0.08071	0.031265	0.01969	0.014235	0.01123	0.010865	0.011555	0.010325		
PLIF	0.008875	0.161965	0.221095	0.18537	0.15114	0.12344	0.091145	0.046655	0.010315		
TLIF	0.014635	0.094455	0.18274	0.18404	0.16586	0.14264	0.111765	0.07465	0.029215		
XLIF	0.04557	0.15975	0.09698	0.089985	0.08005	0.078495	0.09326	0.137885	0.218025		
Leg pain											
	[1]	[2]	[3]	[4]	[5]	[6]	[7]	[8]	[9]		
Endo-PLIF	0.02568	0.107315	0.158385	0.161815	0.14727	0.12521	0.11036	0.104895	0.05907		
Endo-TLIF	0.008795	0.03841	0.066625	0.088775	0.11335	0.130235	0.15211	0.23003	0.17167		
MIS-PLIF	0.05019	0.09364	0.077265	0.057535	0.051925	0.050215	0.058755	0.09783	0.462645		
MIS-TLIF	0.00137	0.015805	0.05124	0.11092	0.177355	0.22477	0.22249	0.146655	0.049395		
OLIF	0.05932	0.20541	0.197995	0.135415	0.111145	0.09638	0.083215	0.07043	0.04069		
PLF	0.195615	0.310545	0.12701	0.07572	0.05717	0.050965	0.052	0.07147	0.059505		
PLIF	0.010125	0.06899	0.177445	0.208335	0.178155	0.14739	0.1242	0.07028	0.01508		
TLIF	0.003305	0.028745	0.09169	0.128285	0.1377	0.153795	0.175425	0.17747	0.103585		
XLIF	0.6456	0.13114	0.052345	0.0332	0.02593	0.02104	0.021445	0.03094	0.03836		
JOA											
	[1]	[2]	[3]								
Endo-TLIF	0.490305	0.205415	0.30428								
MIS-TLIF	0.368985	0.512615	0.1184								
TLIF	0.14071	0.28197	0.57732								
ODI											
	[1]	[2]	[3]	[4]	[5]	[6]	[7]	[8]	[9]	[10]	
circumferential	0.012905	0.020395	0.009095	0.00925	0.01043	0.0095	0.01038	0.02843	0.10788	0.781735	
Endo-PLIF	0.038025	0.08047	0.284105	0.33147	0.18436	0.062835	0.01534	0.00281	0.00053	0.000055	
Endo-TLIF	0.01793	0.03038	0.078065	0.16906	0.19695	0.180195	0.17457	0.10908	0.037085	0.006685	
MIS-PLIF	0.09601	0.043025	0.040705	0.04754	0.046135	0.038705	0.085265	0.254355	0.22253	0.12573	
MIS-TLIF	0	0.000115	0.00394	0.108955	0.300385	0.32916	0.182765	0.061225	0.01232	0.001135	
OLIF	0.37085	0.32351	0.17708	0.081645	0.03211	0.01043	0.003135	0.00093	0.00027	0.00004	
PLF	0.121685	0.045395	0.033195	0.03694	0.034695	0.028275	0.055385	0.174405	0.44393	0.026095	
PLIF	0	0.00004	0.000415	0.011195	0.093645	0.283595	0.354385	0.193835	0.05469	0.0082	
TLIF	0.115625	0.38885	0.30396	0.134865	0.04335	0.01074	0.002205	0.000385	0.00001	0.00001	
XLIF	0.22697	0.06782	0.06944	0.06908	0.05794	0.046565	0.11657	0.174545	0.120755	0.050315	
Complications											
	[1]	[2]	[3]	[4]	[5]	[6]					
Endo-PLIF	0.07022	0.102915	0.079355	0.064425	0.119205	0.56388					
Endo-TLIF	0.12124	0.23003	0.13376	0.10084	0.207775	0.206355					
MIS-TLIF	0.00002	0.088165	0.250025	0.34815	0.25233	0.06131					
OLIF	0.053895	0.36249	0.317165	0.184275	0.073865	0.00831					
TLIF	0.000225	0.013795	0.18328	0.2965	0.34608	0.16012					
XLIF	0.7544	0.202605	0.036415	0.00581	0.000745	0.000025					
Reoperation											
	[1]	[2]	[3]	[4]	[5]	[6]	[7]	[8]			
circumferential	0.083385	0.0472	0.043385	0.0486	0.060635	0.104225	0.232955	0.379615			
Endo-TLIF	0.295445	0.123495	0.11959	0.1021	0.111135	0.097505	0.06699	0.08374			
MIS-TLIF	0.000645	0.0199	0.15479	0.32994	0.255625	0.14121	0.07977	0.01812			
OLIF	0.359205	0.256415	0.157575	0.097745	0.067535	0.035885	0.01727	0.00837			
PLF	0.005825	0.02027	0.030955	0.041125	0.06285	0.11687	0.38219	0.339915			
PLIF	0.07224	0.094405	0.12123	0.142595	0.177305	0.2905	0.085685	0.01604			
TLIF	0.161715	0.39622	0.290995	0.10056	0.038155	0.01144	0.00088	0.000035			
XLIF	0.02154	0.042095	0.08148	0.137335	0.22676	0.202365	0.13426	0.154165			
Fusion											
	[1]	[2]	[3]	[4]	[5]	[6]	[7]	[8]	[9]	[10]	[11]
circumferential	0.42092	0.114155	0.066965	0.05859	0.043385	0.04042	0.04054	0.067445	0.090795	0.056105	0.00068
Endo-PLIF	0.000485	0.001905	0.004135	0.008695	0.017145	0.03009	0.053705	0.120555	0.186225	0.56657	0.01049
Endo-TLIF	0.002965	0.01605	0.037545	0.07686	0.13066	0.177695	0.188065	0.18767	0.138345	0.0437	0.000445
MIS-PLF	0.000045	0.000205	0.000505	0.000525	0.000835	0.000995	0.00099	0.00095	0.001775	0.00673	0.986445
MIS-PLIF	0.103425	0.15843	0.14741	0.130755	0.10518	0.087745	0.08842	0.07732	0.068665	0.03234	0.00031
MIS-TLIF	0.000045	0.003075	0.02144	0.072635	0.10886	0.150095	0.2054	0.236315	0.17634	0.02546	0.000335
OLIF	0.060925	0.11394	0.120315	0.109725	0.11287	0.11604	0.1082	0.092865	0.10509	0.059005	0.001025
PLF	0.01377	0.094445	0.083205	0.08867	0.086105	0.06746	0.07241	0.09623	0.19329	0.20421	0.000205
PLIF	0.04941	0.151035	0.207765	0.181745	0.135675	0.110575	0.089195	0.053625	0.01886	0.002105	0.00001
TLIF	0.024605	0.114055	0.168445	0.164475	0.16246	0.169825	0.1246	0.05426	0.015065	0.002175	0.000035
XLIF	0.323405	0.232705	0.14227	0.107325	0.096825	0.04906	0.028475	0.012765	0.00555	0.0016	0.00002

*LSS* lumbar spinal stenosis, *JOA* Japanese Orthopaedic Association, *ODI* Oswestry Disability Index, *PLF* posterolateral lumbar fusion, *PLIF* posterior lumbar interbody fusion, *TLIF* transforaminal lumbar interbody fusion, *MIS-PLF* minimally invasive posterolateral lumbar fusion, *MIS-PLIF* minimally invasive posterior lumbar interbody fusion, *MIS-TLIF* minimally invasive transforaminal lumbar interbody fusion, *XLIF* extreme lateral interbody fusion, *OLIF* oblique lumbar interbody fusion, *Endo-PILF* percutaneous endoscopic posterior lumbar interbody fusion, *Endo-TILF* percutaneous endoscopic transforaminal lumbar interbody fusion

### Low back pain

Nine fusion approaches were evaluated for the treatment of low back pain in 15 studies of 1,430 patients: Endo-PLIF, Endo-TLIF, MIS-PLIF, MIS-TLIF, OLIF, PLF, PLIF, TLIF, and XLIF (Fig. 2b). The forest plot (Fig. 3b) and league table (Table 1) presented no significant differences in low back pain between these fusion approaches. As suggested by the rank probabilities, Endo-PLIF had the greatest likelihood to be the optimal technique for low back pain, followed sequentially by MIS-TLIF, MIS-PLIF, XLIF, Endo-TLIF, TLIF, OLIF, PLIF, and PLF (Table 2).

### Leg pain

Fourteen studies with 1,324 patients were eligible for leg pain assessment, and 9 fusion approaches were compared: Endo-PLIF, Endo-TLIF, MIS-PLIF, MIS-TLIF, OLIF, PLF, PLIF, TLIF, and XLIF (Fig. 2c). No significant differences were identified by the forest plot (Fig. 3c) and league table (Table 1). From the rank probabilities, MIS-PLIF was ranked the most effective technique concerning leg pain, followed by Endo-TLIF, MIS-TLIF, TLIF, Endo-PLIF, PLIF, OLIF, PLF, and XLIF (Table 2).

## Network meta-analysis for JOA scores

**Fig. 4:**
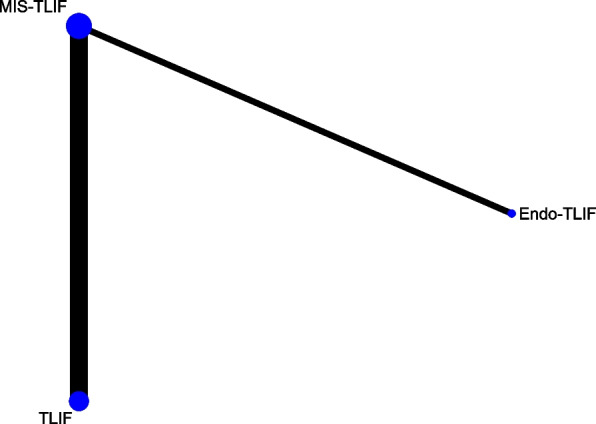
Network plot of different lumbar fusion techniques for JOA scores in LSS. JOA, Japanese Orthopaedic Association; LSS, lumbar spinal stenosis; TLIF, transforaminal lumbar interbody fusion; MIS-TLIF, minimally invasive transforaminal lumbar interbody fusion; Endo-TLIF, percutaneous endoscopic transforaminal lumbar interbody fusion

**Fig. 5:**
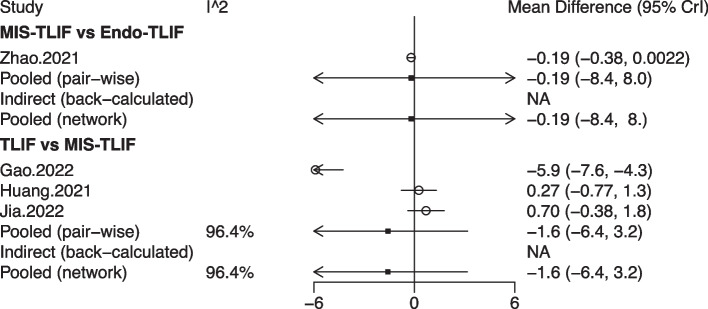
Forest plot of different lumbar fusion techniques for JOA scores in LSS. JOA, Japanese Orthopaedic Association; LSS, lumbar spinal stenosis; TLIF, transforaminal lumbar interbody fusion; MIS-TLIF, minimally invasive transforaminal lumbar interbody fusion; Endo-TLIF, percutaneous endoscopic transforaminal lumbar interbody fusion; CrI, credibility interval

Data on JOA scores were obtained from 4 studies of 320 patients, encompassing 3 fusion techniques: Endo-TLIF, MIS-TLIF and TLIF (Fig. 4). The forest plot (Fig. 5) and league table (Table 1) indicated no significant differences between the fusion techniques. The rank probabilities suggested that as regards JOA scores, Endo-TLIF had the maximum probability to be the best technique, followed by MIS-TLIF and TLIF (Table 2).

## Network meta-analysis for ODI scores

**Fig. 6:**
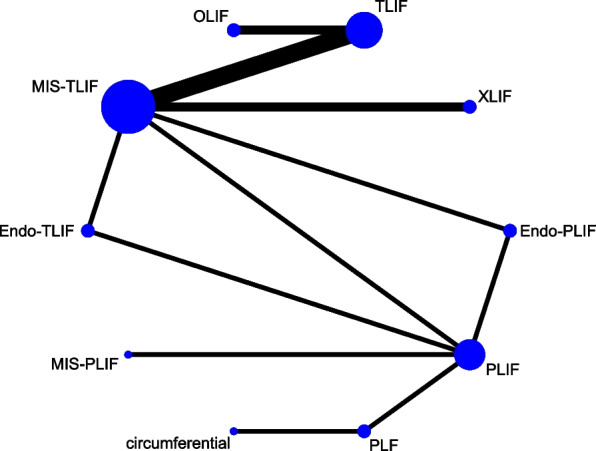
Network plot of different lumbar fusion techniques for ODI scores in LSS. ODI, Oswestry Disability Index; LSS, lumbar spinal stenosis; PLF, posterolateral lumbar fusion; PLIF, posterior lumbar interbody fusion; TLIF, transforaminal lumbar interbody fusion; MIS-PLIF, minimally invasive posterior lumbar interbody fusion; MIS-TLIF, minimally invasive transforaminal lumbar interbody fusion; XLIF, extreme lateral interbody fusion; OLIF, oblique lumbar interbody fusion; Endo-PLIF, percutaneous endoscopic posterior lumbar interbody fusion; Endo-TLIF, percutaneous endoscopic transforaminal lumbar interbody fusion

**Fig. 7:**
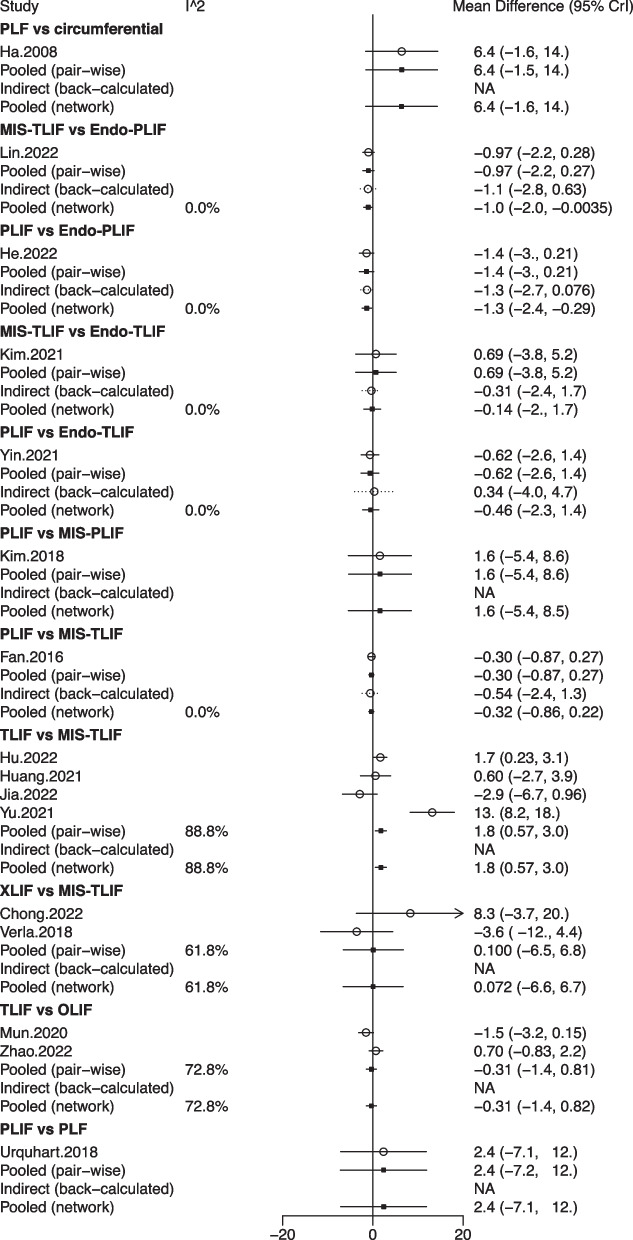
Forest plot of different lumbar fusion techniques for ODI scores in LSS. ODI, Oswestry Disability Index; LSS, lumbar spinal stenosis; PLF, posterolateral lumbar fusion; PLIF, posterior lumbar interbody fusion; TLIF, transforaminal lumbar interbody fusion; MIS-PLIF, minimally invasive posterior lumbar interbody fusion; MIS-TLIF, minimally invasive transforaminal lumbar interbody fusion; XLIF, extreme lateral interbody fusion; OLIF, oblique lumbar interbody fusion; Endo-PLIF, percutaneous endoscopic posterior lumbar interbody fusion; Endo-TLIF, percutaneous endoscopic transforaminal lumbar interbody fusion; CrI, credibility interval

ODI scores were investigated by 16 studies with 1,328 patients, and 10 fusion methods were evaluated: circumferential fusion, Endo-PLIF, Endo-TLIF, MIS-PLIF, MIS-TLIF, OLIF, PLF, PLIF, TLIF, and XLIF (Fig. 6). Based on the forest plot, the ODI score after TLIF was significantly higher than that after MIS-TLIF (pooled WMD = 1.80, 95%CrI: 0.57, 3.00) (Fig. 7). As exhibited by the league table, the ODI score after MIS-TLIF (pooled WMD = -1.01, 95%CrI: -2.01, -0.004) or PLIF (pooled WMD = -1.33, 95%CrI: -2.37, -0.29) was significantly lower than that after Endo-PLIF. Patients undergoing OLIF (pooled WMD = 2.09, 95%CrI: 0.43, 3.74) or TLIF (pooled WMD = 1.78, 95%CrI: 0.57, 3.00) had a significantly higher ODI score than those undergoing MIS-TLIF. PLIF was associated with a significantly decreased ODI score versus OLIF (pooled WMD = -2.41, 95%CrI: -4.15, -0.66). The ODI score following TLIF was significantly higher than that after PLIF (pooled WMD = 2.10, 95%CrI: 0.76, 3.43) (Table 1). The rank probabilities showed that circumferential fusion was most likely to be the optimum technique concerning ODI scores, followed by PLF, MIS-PLIF, PLIF, MIS-TLIF, Endo-TLIF, XLIF, Endo-PLIF, TLIF, and OLIF (Table 2).

## Network meta-analysis for complications

**Fig. 8:**
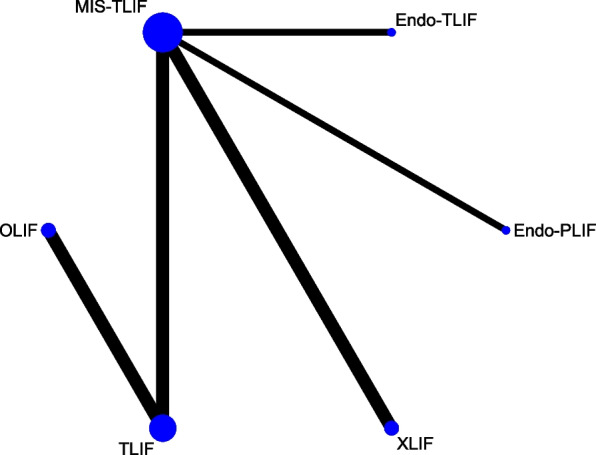
Network plot of different lumbar fusion techniques for complications in LSS. LSS, lumbar spinal stenosis; TLIF, transforaminal lumbar interbody fusion; MIS-TLIF, minimally invasive transforaminal lumbar interbody fusion; XLIF, extreme lateral interbody fusion; OLIF, oblique lumbar interbody fusion; Endo-PLIF, percutaneous endoscopic posterior lumbar interbody fusion; Endo-TLIF, percutaneous endoscopic transforaminal lumbar interbody fusion

**Fig. 9:**
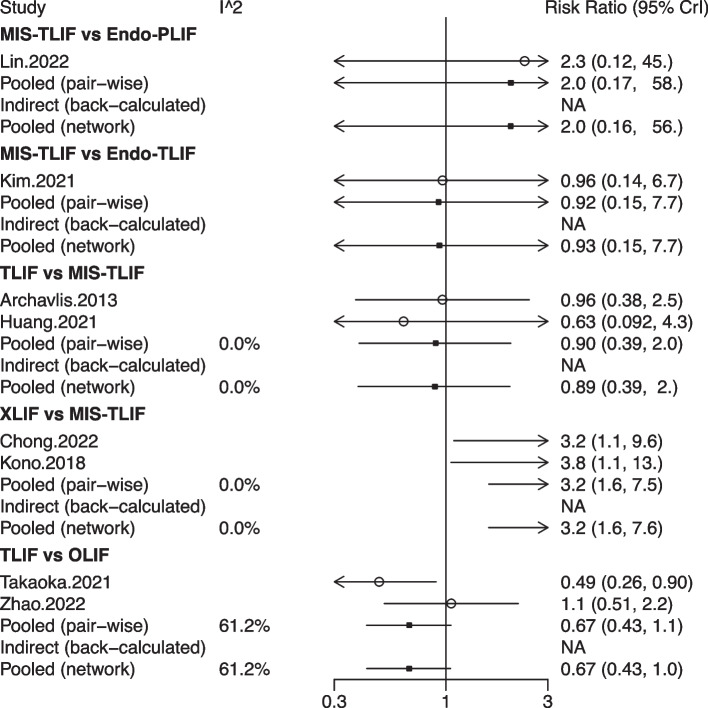
Forest plot of different lumbar fusion techniques for complications in LSS. LSS, lumbar spinal stenosis; TLIF, transforaminal lumbar interbody fusion; MIS-TLIF, minimally invasive transforaminal lumbar interbody fusion; XLIF, extreme lateral interbody fusion; OLIF, oblique lumbar interbody fusion; Endo-PLIF, percutaneous endoscopic posterior lumbar interbody fusion; Endo-TLIF, percutaneous endoscopic transforaminal lumbar interbody fusion; CrI, credibility interval

A total of 8 studies with 620 patients involved 6 fusion techniques (Endo-PLIF, Endo-TLIF, MIS-TLIF, OLIF, TLIF, and XLIF) for complication assessment (Fig. 8). The forest plot demonstrated that the incidence of complications in patients undergoing XLIF was significantly higher than that in patients undergoing MIS-TLIF (pooled RR = 3.80, 95%CrI: 1.10, 13.00) (Fig. 9). According to the league table, compared with MIS-TLIF (pooled RR = 3.19, 95%CrI: 1.57, 7.63) or TLIF (pooled RR = 3.59, 95%CrI: 1.21, 11.80), XLIF was associated with a significantly increased incidence of complications (Table 1). As suggested by the rank probabilities, Endo-PLIF had the greatest likelihood to be the best technique for complications, followed by TLIF, MIS-TLIF, Endo-TLIF, OLIF, and XLIF (Table 2).

## Network meta-analysis for reoperation

**Fig. 10:**
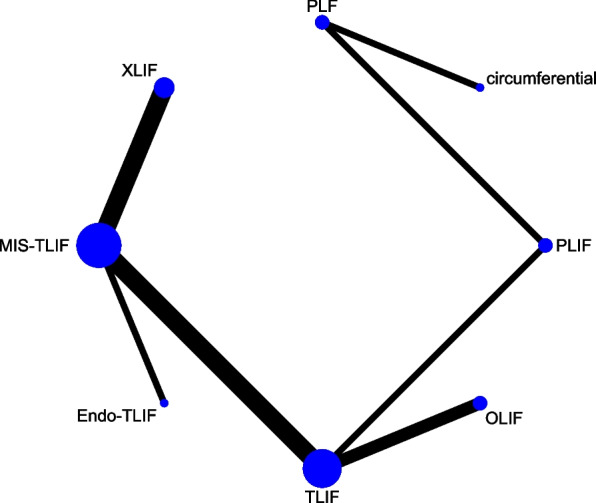
Network plot of different lumbar fusion techniques for reoperation in LSS. LSS, lumbar spinal stenosis; PLF, posterolateral lumbar fusion; PLIF, posterior lumbar interbody fusion; TLIF, transforaminal lumbar interbody fusion; MIS-TLIF, minimally invasive transforaminal lumbar interbody fusion; XLIF, extreme lateral interbody fusion; OLIF, oblique lumbar interbody fusion; Endo-TLIF, percutaneous endoscopic transforaminal lumbar interbody fusion

**Fig. 11:**
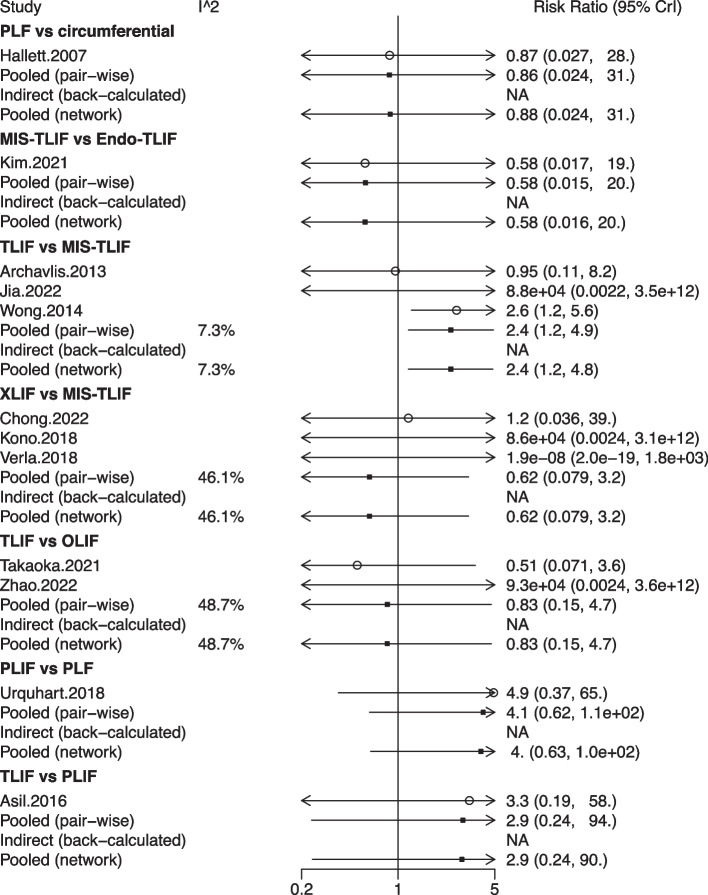
Forest plot of different lumbar fusion techniques for reoperation in LSS. LSS, lumbar spinal stenosis; PLF, posterolateral lumbar fusion; PLIF, posterior lumbar interbody fusion; TLIF, transforaminal lumbar interbody fusion; MIS-TLIF, minimally invasive transforaminal lumbar interbody fusion; XLIF, extreme lateral interbody fusion; OLIF, oblique lumbar interbody fusion; Endo-TLIF, percutaneous endoscopic transforaminal lumbar interbody fusion; CrI, credibility interval

As for reoperation, 12 studies of 1,026 patients were included for network meta-analysis. Comparisons were carried out among 8 fusion methods: circumferential fusion, Endo-TLIF, MIS-TLIF, OLIF, PLF, PLIF, TLIF, and XLIF (Fig. 10). TLIF was associated with a significantly higher incidence of reoperation relative to MIS-TLIF (pooled RR = 2.40, 95%CrI: 1.20, 4.90), as presented by the forest plot (Fig. 11). The league table showed that compared with the incidence of reoperation after MIS-TLIF, that after TLIF was significantly greater (pooled RR = 2.41, 95%CrI: 1.19, 4.84) (Table 1). The rank probabilities illustrated that for reoperation, PLF was most likely to be the optimal method, followed by circumferential fusion, XLIF, MIS-TLIF, PLIF, Endo-TLIF, TLIF, and OLIF (Table 2).

## Network meta-analysis for fusion

**Fig. 12:**
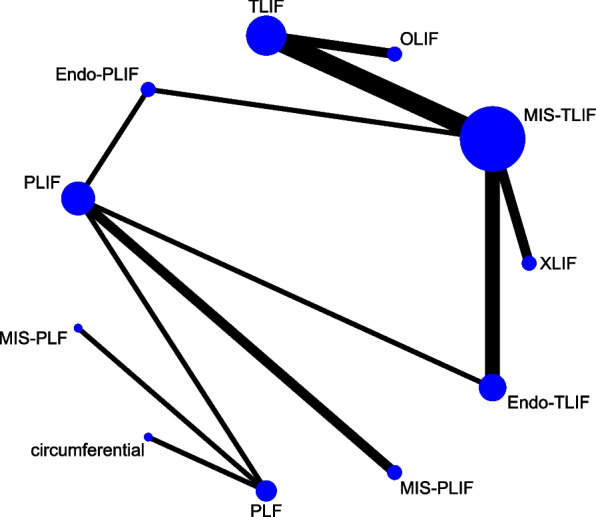
Network plot of different lumbar fusion techniques for fusion in LSS. LSS, lumbar spinal stenosis; PLF, posterolateral lumbar fusion; PLIF, posterior lumbar interbody fusion; TLIF, transforaminal lumbar interbody fusion; MIS-PLF, minimally invasive posterolateral lumbar fusion; MIS-PLIF, minimally invasive posterior lumbar interbody fusion; MIS-TLIF, minimally invasive transforaminal lumbar interbody fusion; XLIF, extreme lateral interbody fusion; OLIF, oblique lumbar interbody fusion; Endo-PLIF, percutaneous endoscopic posterior lumbar interbody fusion; Endo-TLIF, percutaneous endoscopic transforaminal lumbar interbody fusion

**Fig. 13:**
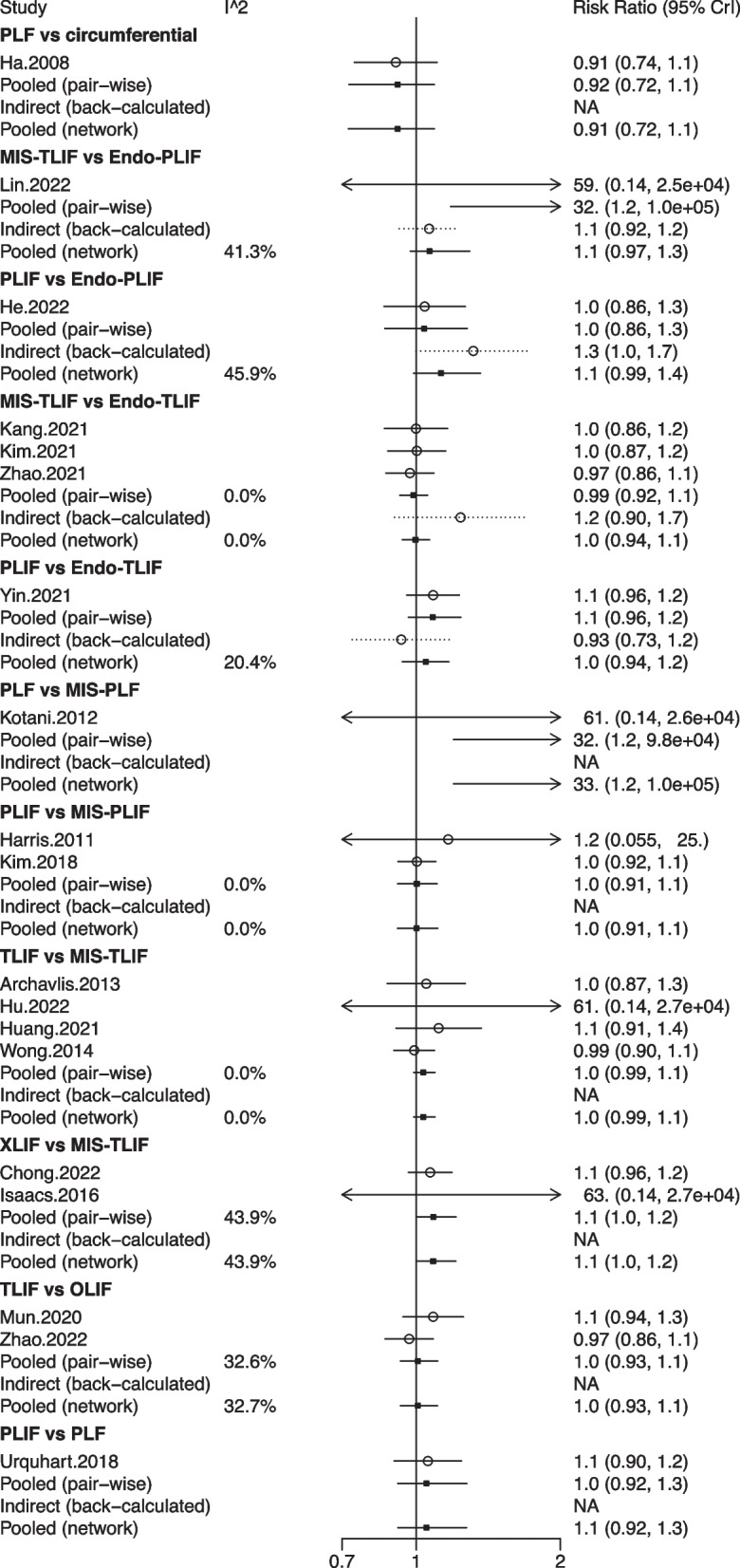
Forest plot of different lumbar fusion techniques for fusion in LSS. LSS, lumbar spinal stenosis; PLF, posterolateral lumbar fusion; PLIF, posterior lumbar interbody fusion; TLIF, transforaminal lumbar interbody fusion; MIS-PLF, minimally invasive posterolateral lumbar fusion; MIS-PLIF, minimally invasive posterior lumbar interbody fusion; MIS-TLIF, minimally invasive transforaminal lumbar interbody fusion; XLIF, extreme lateral interbody fusion; OLIF, oblique lumbar interbody fusion; Endo-PLIF, percutaneous endoscopic posterior lumbar interbody fusion; Endo-TLIF, percutaneous endoscopic transforaminal lumbar interbody fusion; CrI, credibility interval

The fusion rate was evaluated in 19 studies with 1,704 patients, and 11 fusion techniques were compared: circumferential fusion, Endo-PLIF, Endo-TLIF, MIS-PIF, MIS-PLIF, MIS-TLIF, OLIF, PLF, PLIF, TLIF, and XLIF (Fig. 12). The forest plot exhibited a significant difference in the fusion rate between the XLIF and MIS-TLIF groups (pooled RR = 1.10, 95%CrI: 1.00, 1.20) (Fig. 13). The league table demonstrated that the fusion rate after MIS-PLF was significantly lower than that after circumferential fusion (pooled RR = 0.03, 95%CrI: 0.00, 0.76), Endo-PLIF (pooled RR = 0.03, 95%CrI: 0.00, 0.90), or Endo-TLIF (pooled RR = 0.03, 95%CrI: 0.00, 0.83). A significantly elevated fusion rate was shown in patients treated with XLIF versus those treated with Endo-PLIF (pooled RR = 1.17, 95%CrI: 1.02, 1.44) or MIS-TLIF (pooled RR = 1.09, 95%CrI: 1.01, 1.21) (Table 1). According to the rank probabilities, XLIF had the highest possibility to be the most effective technique regarding the fusion rate, followed by circumferential fusion, PLIF, MIS-PLIF, TLIF, OLIF, Endo-TLIF, PLF, MIS-TLIF, Endo-PLIF, and MIS-PLF in sequence (Table 2).

## Discussion

To the best of our knowledge, this network meta-analysis comprehensively evaluated different lumbar fusion techniques for pain, JOA, ODI, complications, reoperation, and fusion among patients with LSS for the first time. The findings demonstrated that minimally invasive fusion techniques may be effective for LSS patients, in terms of pain, JOA and complications, suggesting that minimally invasive approaches may be safe and feasible in the treatment of LSS.

A meta-analysis by Gagliardi et al. [48] compared the impacts of indirect (ALIF, OLIF, and lateral LIF) and direct (TLIF and PLIF) decompression and fusion approaches on postoperative pain and disability in patients with LSS and instability, and showed that indirect and direct approaches had comparable effects. PLIF, PLF, MIS-PLIF, TLIF, and MIS-TLIF were subject to a network meta-analysis for patients with spondylolisthesis [49], and another network meta-analysis simultaneously evaluated the efficacy of PLF, PLIF, TLIF, MIS-TLIF, XLIF, and circumferential fusion in spondylolisthesis [50]. At present, no study has evaluated and ranked the influences of various fusion techniques in patients with LSS via a Bayesian network meta-analysis. The current Bayesian network meta-analysis filled this research gap, and paid attention to 11 individual fusion techniques for LSS patients, and these techniques can be divided into traditional and minimally invasive techniques. As traditional techniques, PLF, PLIF and TLIF are widely accepted treatments in LSS. Said et al. [51] showed that PLF and PLIF had similar complication rates, operation time and blood loss, while PLIF exhibited a greater rate of fusion. TLIF was reported to reduce the possible complications of other techniques, including the transabdominal method or PLIF, but gain similar clinical outcomes to PLIF [52]. As surgical tools develop and advance, multifarious minimally invasive spinal operations have emerged and been enhanced, including indirect decompression approaches using interspinous instrumentation and direct decompression approaches, like microscopic or endoscopic spinal surgery [53]. In this study, we made comparisons between different kinds of traditional and minimally invasive techniques to review and rank their effects in LSS.

In terms of pain, we found that LSS patients undergoing minimally invasive fusion may have less pain, low back pain, and leg pain than those undergoing traditional fusion. Low back pain and leg pain are classical symptoms, affecting the quality of life, which may be attributed to nerve root compression and associated instability [53]. Minimally invasive techniques include MIS- and Endo-fusion approaches, which can improve surgical visualization, reduce tissue trauma and normal structure damage, and lessen postoperative pain [54–56]. As regards functional status evaluated by JOA and ODI scores, minimally invasive operations (Endo-TLIF and MIS-TLIF) may exhibit more favorable impacts than the traditional one (TLIF) according to the JOA score, while based on the ODI score, minimally invasive techniques (e.g. Endo-PLIF, Endo-TLIF) may not have better efficacy in general. Hoffmann and Frank [57] showed patients undergoing MIS-TLIF had notably lower ODI scores than those undergoing TLIF, which was partially consistent with our findings. More studies are warranted to verify these results. With respect to complications, minimally invasive techniques may be generally non-inferior to traditional techniques, with Endo-PLIF having the highest likelihood to be the most effective approach. Minimally invasive posterior methods have been developed to reduce relevant complications [58]. Concerning reoperation, patients after traditional fusion techniques may not have a superior reoperation rate to those after minimally invasive techniques. Another meta-analysis reported that minimally invasive decompression was associated with reduced reoperation and fusion rates, decreased slip progression, and increased patient satisfaction versus open surgery in patients with LSS and degenerative spondylolisthesis [59]. For experienced clinicians, most patients can safely obtain appropriate decompression through minimally invasive approaches. Regarding the fusion rate, compared with traditional surgery, minimally invasive surgery may not gain the upper hand. Wu et al. [60] showed relatively high and comparable fusion rates in patients with degenerative disease who underwent TLIF and MIS-TLIF. Besides surgical methods, other factors may influence the success of fusion, such as patient age, comorbidities, personal lifestyles, and fusion levels. Due to insufficient reporting of the included studies, these factors could not be taken into account in this analysis, which underscores future research to assess our results and indicates the clinical importance of improved reporting in studies.

Through comprehensive analysis of different fusion techniques in patients with LSS, minimally invasive techniques may be effective and feasible for LSS management. Combining direct and indirect clinical evidence can yield robust results, which may act as a clinical decision-making guidance in the control and treatment of LSS. Minimally invasive fusion techniques might be considered by clinicians to improve pain and functional status and reduce the incidence of complications in patients with LSS. Besides, this network meta-analysis adopted a Bayesian approach. Compared with a frequentist approach, a Bayesian approach can not only effectively integrate data and flexibly build models, but also use the obtained posterior probability to rank all interventions participating in the comparison and distinguish comparative advantages and disadvantages, while a frequentist method can only rely on the effect size and its 95%CI obtained by pairwise comparison in ranking; and since a frequentist approach uses the maximum likelihood method in parameter estimation, it is prone to instability and biased results, while a Bayesian approach does not have this problem, so its estimated values are more accurate than those of a frequentist approach [19]. Of note, minimally invasive techniques were related to a steep learning curve, and surgeons should not expect to master these techniques in the first few cases [61]. There were several limitations in this study. First, heterogeneity in the study population may have affected the reliability of the results. For example, most LSS patients also had other degenerative diseases such as spondylolisthesis, lumbar instability, and lumbar disc herniation; some included studies reported single-level fusion, and some involved multi-level fusion. Studies on LSS patients with fusion levels ≤ 3 and comparing at least two of different lumbar fusion techniques for spinal level L3-L5 were included for this network meta-analysis. We also tried to make the severity of LSS and previous lumbar spine surgery equivalent among patients based on the data from the included studies, but only one study reported the severity of LSS, and many studies did not report whether patients underwent previous surgery, which made it difficult to equate patients in these aspects. Future studies should improve and standardize the reporting of LSS patient condition. Second, studies on patients with lumber degenerative disease and lateral lumbar interbody fusion (LLIF) were excluded due to no clear classification. The accuracy of the results may have been affected. Third, some fusion methods, such as MIS-PLF and circumferential fusion, were assessed by a small number of studies and a small sample size, which may have influenced the stability of the results. Finally, only English publications were included for analysis, which may have led to language bias and limited the generalizability of the results.

## Conclusion

Compared with traditional techniques, minimally invasive fusion techniques may be effective and feasible for LSS treatment, considering pain, JOA, and complications. Additional prospective research is required to consolidate our findings.

### Supplementary Information

**Additional file 1: Supplementary Table 1.** Baseline characteristics of the included studies. 

## Data Availability

The datasets used and/or analyzed during the current study are available from the corresponding author on reasonable request.

## Abbreviations

LSS: Lumbar spinal stenosis
PLF: Posterolateral lumbar fusion
PLIF: Posterior lumbar interbody fusion
TLIF: Transforaminal lumbar interbody fusion
MIS: Minimally invasive surgery
Endo-LIF: Endoscopic lumbar interbody fusion
OLIF: Oblique lumbar interbody fusion
MIS-TLIF: Minimally invasive transforaminal lumbar interbody fusion
MIS-PLIF: Minimally invasive posterior lumbar interbody fusion
MIS-PLF: Minimally invasive posterolateral lumbar fusion
XLIF: Extreme lateral interbody fusion
Endo-PLIF: Endoscopic posterior lumbar interbody fusion
Endo-TLIF: Endoscopic transforaminal lumbar interbody fusion
JOA: Japanese Orthopaedic Association
ODI: Oswestry Disability Index
RCTs: Randomized controlled trials
BMI: Body mass index
FU: Follow-up time
QA: Quality assessment
