# Supplementary material for: Different lumbar fusion techniques for lumbar spinal stenosis: a Bayesian network meta-analysis
Source: BMC Surg. 2023 Nov 15;23:345. doi: 10.1186/s12893-023-02242-w (PMC10652640; doi:10.1186/s12893-023-02242-w)
Supplement: Supplementary file 1 — Additional file 1: Supplementary Table 1. Baseline characteristics of the included studies. [file 12893_2023_2242_MOESM1_ESM.docx]

Supplementary Table 1 Baseline characteristics of the included studies

| Author | Year | Country | Study design | Population | Group | N | Sex (M/F) | Age (years) | BMI (kg/m^2^) | Spinal level | Fusion level | FU (months) | QA | Outcome |
| --- | --- | --- | --- | --- | --- | --- | --- | --- | --- | --- | --- | --- | --- | --- |
| Chong | 2022 | Singapore | Retrospective cohort | LSS ± spondylolisthesis | XLIF | 24 | 9/15 | 68.4± 6.8 | 25.5 ± 3.7 | L3-4 and L4-5 | 2 level | 24 | 8 | Pain (leg, back), ODI, complications, reoperation, fusion |
|  |  |  |  |  | MIS-TLIF | 29 | 9/20 | 65.3± 9.3 | 26.5 ± 4.3 |  |  |  |  |  |
| Gao | 2022 | China | Cohort | LSS | MIS-TLIF | 31 | 12/19 | 65.7 (56-78) | - | L4-5 16, L5-S1 10, L4-S1 5 | 1-level 26, 2-level 5 | 12 | 6 | Pain, JOA |
|  |  |  |  |  | TLIF | 29 | 13/16 |  |  | L4-5 14, L5-S1 9, L4-S1 6 | 1-level 23, 2-level 6 |  |  |  |
| He | 2022 | China | Retrospective cohort | LSS + spondylolisthesis | Endo-PLIF | 28 | 14/14 | 59.8 ± 10.9 | 24.6 ± 2.0 | L4-5 20, L5-S1 8 | 1-level | 18.4 ± 1.3 | 8 | Pain (leg, back), ODI, fusion |
|  |  |  |  |  | PLIF | 28 | 13/15 | 54.2 ± 10.3 | 24.4 ± 3.5 | L4-5 17, L5-S1 11 |  | 18.9 ± 1.7 |  |  |
| Hu | 2022 | China | Retrospective cohort | LSS ± spondylolisthesis | MIS-TLIF | 52 | 28/24 | 64.18 ± 8.17 | 32.41 ± 3.87 | L3-5 16, L4-S1 36 | 2-level | 14.7±2.1 | 7 | Pain (leg, back), ODI, fusion |
|  |  |  |  |  | TLIF | 60 | 34/26 | 66.24 ± 7.16 | 33.74 ± 4.15 | L3-5 22, L4-S1 38 |  |  |  |  |
| Jia | 2022 | China | Retrospective cohort | LSS ± spondylolisthesis | MIS-TLIF | 57 | 22/35 | 57.7 ± 10.0 | - | L4-5 | 1-level | 64.4 ± 3.7 | 6 | Pain (leg, back), JOA, ODI, reoperation |
|  |  |  |  |  | TLIF | 64 | 20/44 | 56.0 ± 9.1 |  |  |  | 64.0 ± 3.7 |  |  |
| Lin | 2022 | China | Retrospective cohort | LSS | Endo-PLIF | 41 | 15/26 | 61.85 ± 10.45 | 25.11 ± 2.58 | L3-4 3, L4-5 24, L5-S1 14 | 1-level | 14.13 ± 3.91 | 7 | Pain (leg, back), ODI, complications, fusion |
|  |  |  |  |  | MIS-TLIF | 48 | 18/30 | 62.98 ± 10.52 | 24.47 ± 2.45 | L3-4 1, L4-5 33, L5-S1 14 |  | 13.66 ± 3.67 |  |  |
| Zhao | 2022 | China | Retrospective cohort | LSS + degenerative dynamic instability | OLIF | 46 | 20/26 | 61.7 ± 9.1 | 25.2 ± 3.7 | L4-5 | 1-level | 49.9±1.6 | 6 | Pain (leg, back), ODI, complications, reoperation, fusion |
|  |  |  |  |  | TLIF | 52 | 21/31 | 63.8 ± 10.8 | 24.9 ± 3.3 |  |  | 51.2±2.5 |  |  |
| Huang | 2021 | China | Retrospective cohort | LSS + LDH | MIS-TLIF | 31 | 16/15 | 72.26 ± 3.43 | 24.85 ± 2.93 | L3-4 1, L4-5 24, L5-S1 6 | 1-level | 26.2 (20-36) | 7 | Pain, JOA, ODI, complications, fusion |
|  |  |  |  |  | TLIF | 30 | 20/10 | 71.33 ± 3.41 | 24.87 ± 2.10 | L3-4 2, L4-5 23, L5-S1 5 |  |  |  |  |
| Kang | 2021 | Korea | Retrospective cohort | LSS ± spondylolisthesis/segmental instability | Endo-TLIF | 47 | 17/30 | 66.87 ± 10.41 | 25.32 ± 3.15 | L2-3 4, L3-4 7, L4-5 34, L5–S1 20 | 1-level 29, 2-level 18 | 14.5 ± 2.3 | 6 | Fusion |
|  |  |  |  |  | MIS-TLIF | 32 | 17/15 | 66.38 ± 9.45 | 26.23 ± 3.26 | L2-3 1, L3-4 9, L4-5 22, L5–S1 11 | 1-level 21, 2-level 11 | 15.78 ± 3.16 |  |  |
| Kim | 2021 | Korea | Retrospective cohort | LSS + spondylolisthesis | Endo-TLIF | 32 | 17/15 | 70.5 ± 8.26 | - | L2-3 1, L3-4 3, L4-5 20, L5–S1 8 | 1-level | 27.2 ± 5.4 | 6 | Pain (leg, back), ODI, complications, reoperation, fusion |
|  |  |  |  |  | MIS-TLIF | 55 | 25/30 | 67.3 ± 10.7 |  | L3-4 2, L4-5 46, L5–S1 7 |  | 31.5 ± 7.3 |  |  |
| Takaoka | 2021 | Japan | Retrospective cohort | LSS + DS | OLIF | 65 | 27/38 | 66 ± 12 | - | L3-4 13, L4-5 53 | 1-level | 64.0 ± 16.2 | 7 | Pain (leg, back), complications, reoperation |
|  |  |  |  |  | TLIF | 78 | 36/42 | 71 ± 9 |  | L3-4 8, L4-5 70 |  | 53.0 ± 13.0 |  |  |
| Yin | 2021 | China | Prospective cohort | LSS + degenerative instability | Endo-TLIF | 56 | 10/46 | 60.50±9.56 | - | L4-5 | 1-level | 15.33±3.07 | 7 | Pain (leg, back), ODI, fusion |
|  |  |  |  |  | PLIF | 58 | 10/48 | 60.64±7.42 |  |  |  | 15.82±2.95 |  |  |
| Yu | 2021 | China | Retrospective cohort | LSS | MIS-TLIF | 40 | 19/21 | 59.1±12.48 | - | L4-5 23, L5-S1 17 | 1-level | 12 | 7 | Pain (leg, back), ODI |
|  |  |  |  |  | TLIF | 46 | 26/20 | 55.6±9.85 |  | L4-5 27, L5-S1 19 |  |  |  |  |
| Zhao | 2021 | China | Retrospective cohort | LSS ± LDH | Endo-TLIF | 40 | 23/17 | 56.93 ± 1.66 | - | L3-4 7, L4-5 24, L5-S1 9 | - | 30.7 (24-34) | 7 | Pain, JOA, fusion |
|  |  |  |  |  | MIS-TLIF | 38 | 20/18 | 57.01 ± 0.95 |  | L3-4 8, L4-5 22, L5-S1 8 |  |  |  |  |
| Hiyama | 2020 | Japan | Retrospective cohort | LSS + DS | MIS-TLIF | 44 | 27/17 | 66.9 ± 10.3 | - | L3-4 13, L4-5 29, L5–S1 13 | 1-level 33, 2-level 11 | 12.6 ± 5.6 | 7 | Pain (back) |
|  |  |  |  |  | XLIF | 62 | 37/25 | 70.2 ± 8.1 |  | L1-2 2, L2-3 12, L3-4 31, L4-5 41 | 1-level 43, 2-level 14, 3-level 5 |  |  |  |
| Mun | 2020 | Korea | Retrospective cohort | LSS | OLIF | 74 | 20/54 | 64.1±9.3 | - | L5-S1 | - | 12.1 (5–15) | 7 | Pain (leg, back), ODI, fusion |
|  |  |  |  |  | TLIF | 74 | 24/50 | 66.4±10.6 |  |  |  | 22.3 (9–32) |  |  |
| Kim | 2018 | Korea | RCT | LSS | MIS-PLIF | 37 | 19/18 | 65.4 ± 10.4 | 25.9 | L2-3 3, L3-4 6, L4-5 22, L5–S1 11 | 1-level 32, 2-level 5 | 12 | 3 | Pain (leg, back), ODI, fusion |
|  |  |  |  |  | PLIF | 41 | 22/19 | 66.0 ± 8.6 | 25.3 | L2-3 2, L3-4 8, L4-5 23, L5–S1 12 | 1-level 37, 2-level 4 |  |  |  |
| Kono | 2018 | Japan | Retrospective cohort | LSS + DS | XLIF | 20 | 10/10 | 69.9±7.5 | - | L3-4 6, L4-5 14 | 1-level | ≥12 | 6 | Complications, reoperation |
|  |  |  |  |  | MIS-TLIF | 20 | 7/13 | 66.2±8 |  | L3-4 3, L4-5 17 |  |  |  |  |
| Urquhart | 2018 | Canada | Retrospective cohort | LSS + DS | PLF | 29 | 16/13 | 63.1 ± 9.6 | 27.6 ± 4.1 | L3-4 7, L4-5 22 | 1-level | 23 ± 4 | 7 | Pain (leg, back), ODI, reoperation, fusion |
|  |  |  |  |  | PLIF | 58 | 21/37 | 65.7 ± 9.3 | 28.9 ± 5.7 | L3-4 5, L4-5 53 |  |  |  |  |
| Verla | 2018 | USA | Retrospective cohort | LSS + spondylolisthesis | MIS-TLIF | 29 | 9/20 | 59.9 ± 12.5 | 29.9 ± 4.9 | L3-4 2, L4-5 27 | 1-level | 6.7 ± 4.9 | 7 | Pain, ODI, reoperation |
|  |  |  |  |  | XLIF | 17 | 10/7 | 56.1 ± 9.9 | 27.8 ± 3.9 | L1-2 2, L2-3 3, L3-4 6, L4-5 6 |  | 8.1 ± 3.8 |  |  |
| Asil | 2016 | Turkey | Retrospective cohort | LSS + spondylolisthesis | PLIF | 41 | 12/29 | 55.76 ± 7.78 | - | L3-4 5, L4-5 24, L5-S1 12 | 1-level | 12 | 6 | Reoperation |
|  |  |  |  |  | TLIF | 33 | 8/25 | 53.76 ± 7.95 |  | L3-4 7, L4-5 18, L5-S1 8 |  |  |  |  |
| Fan | 2016 | China | Retrospective cohort | LSS | MIS-TLIF | 24 | 14/10 | 65.9 | - | L1-4 6, L2-5 10, L3-S1 8 | 1-level 7, 2-level 8, 3-level 9 | 14.2 | 7 | Pain (leg, back), ODI |
|  |  |  |  |  | PLIF | 36 | 17/19 | 64.4 |  | L1-4 8, L2-5 16, L3-S1 12 | 1-level 13, 2-level 9, 3-level 14 | 13.4 |  |  |
| Isaacs | 2016 | USA | Prospective cohort | LSS + DS | XLIF | 29 | 13/16 | 63 | 30.1 | L3-4 12, L4-5 24 | 1-level 22, 2-level 7 | 24 | 7 | Fusion |
|  |  |  |  |  | MIS-TLIF | 26 | 11/15 | 64 | 30.1 | L3-4 5, L4-5 26 | 1-level 23, 2-level 3 |  |  |  |
| Wong | 2014 | USA | Retrospective cohort | LSS ± spondylolisthesis/postlaminectomy instability/DDD | MIS-TLIF | 144 | 61/83 | 61 | - | L2-3 3, L3-4 9, L4-5 43, L5–S1 45 | 1-level 79, 2-level 55 | 45 (34–60) | 6 | Reoperation, fusion |
|  |  |  |  |  | TLIF | 54 | 25/29 | 58 |  | L2-3 2, L3-4 7, L4-5 52, L5–S1 39 | - | 46 (33–58) |  |  |
| Archavlis | 2013 | Germany | Retrospective cohort | LSS + DS + facet joint osteoarthritis | MIS-TLIF | 24 | 10/14 | 67 ± 8 | 28 ± 6 | L3-4 2, L4-5 16, L5-S1 6 | 1-level | 26 (24–29) | 7 | Complications, reoperation, fusion |
|  |  |  |  |  | TLIF | 25 | 8/17 | 68 ± 7 | 27 ± 4 | L3-4 1, L4-5 17, L5-S1 7 |  | 26 (24–30) |  |  |
| Kotani | 2012 | Japan | Prospective cohort | LSS + DS | MIS-PLF | 43 | 14/29 | 63 ± 9 | - | - | 1-level | 32 (24–49) | 7 | Fusion |
|  |  |  |  |  | PLF | 37 | 12/25 | 66 ± 9 |  |  |  | 40 (24–60) |  |  |
| Harris | 2011 | USA | Retrospective cohort | LSS + DS | MIS-PLIF | 30 | 10/20 | 66 | - | L4-5 | 1-level | 12 | 6 | Complications, fusion |
|  |  |  |  |  | PLIF | 21 | 9/12 | 69.1 |  |  |  |  |  |  |
| Ha | 2008 | Korea | Retrospective cohort | LSS + DS | PLF + PLIF | 19 | 11/29 | 57.8 (33-76) | - | L4-5 | 1-level | 53 (24-130) | 6 | ODI, fusion |
|  |  |  |  |  | PLF | 21 |  |  |  |  |  |  |  |  |
| Hallett | 2007 | Scotland | RCT | LSS + DDD | PLF | 16 | 6/10 | 54±9 | - | L3-4 2, L4-5 9, L5–S1 5 | 1-level | 60 | 4 | Reoperation |
|  |  |  |  |  | PLF + TLIF | 14 | 9/5 | 59±9 |  | L3-4 3, L4-5 8, L5–S1 3 |  |  |  |  |

N, sample size; M/F, male/female; BMI, body mass index; FU, follow-up time; QA, quality assessment; DDD, degenerative disc disease; DS, degenerative spondylolisthesis; Endo-PLIF, percutaneous endoscopic posterior lumbar interbody fusion; Endo-TLIF, percutaneous endoscopic transforaminal lumbar interbody fusion; JOA, Japanese orthopaedic association scores; LDH, Lumbar disc herniation; LSS, lumbar spinal stenosis; MIS-PLF, minimally invasive posterolateral lumbar fusion; MIS-PLIF, minimally invasive posterior lumbar interbody fusion; MIS-TLIF, minimally invasive transforaminal lumbar interbody fusion; ODI, oswestry disability index; OLIF, oblique lumbar interbody fusion; PLF, posterolateral lumbar fusion; PLIF, posterior lumbar interbody fusion; PS, pedicle screw; RCT, randomized controlled trial; TLIF, transforaminal lumbar interbody fusion; XLIF, extreme lateral interbody fusion.
